# An investigative study into the influence of a commercially available carbohydrate-protein-electrolyte beverage on short term repeated exercise performance

**DOI:** 10.1186/1550-2783-9-5

**Published:** 2012-03-09

**Authors:** Justin D Roberts, Michael D Tarpey, Lindsy S Kass, Michael G Roberts

**Affiliations:** 1 School of Life Sciences, Division of Sport, Health and Exercise, University of Hertfordshire, College Lane, Hatfield, Hertfordshire, UK

**Keywords:** Carbohydrate, Maltodextrin, Cycling performance, Power output, Oxidation rates

## Abstract

**Background:**

The purpose of this study was to undertake an independent investigation into the effects of ingesting a carbohydrate-protein-electrolyte (CPE) beverage on repeated submaximal and time-trial cycling performance.

**Methods:**

Sixteen recreationally trained males (height: 1.76 ± 0.07 m; weight: 70.05 ± 7.90 kg; VO_2max_: 49.69 ± 4.19 ml.kg^-1^.min^-1^) performed two exercise trials separated by 7 days. Each trial comprised two bouts of 90 minutes exercise separated by a 2 hour recovery period. Each bout comprised 45 minutes exercise on a cycle-ergometer at 60%VO_2max _(ST), followed immediately by a 45 minute performance test (PT). Participants were randomly assigned an 8% CPE beverage or colour/taste matched placebo (PL) prior to each trial. Participants consumed 100 ml of the assigned beverage every 10 minutes during each ST, and 500 ml at 0 and 60 minutes into recovery (total caloric delivery per trial: 617.6 kcal for CPE and12.8 kcal for PL). Mean power output (W), speed (km.hr^-1^) and distance covered (km) were assessed throughout both trials. Expired air was sampled at 10 minute intervals throughout ST. Blood glucose and lactate were assessed during ST and recovery.

**Results:**

Distance covered during ST was significantly reduced with PL by 9.12% (20.18 ± 0.28 km in ST1 v 18.34 ± 0.36 km in ST2; *P *= 0.0001). With CPE, distance covered, power output and average speed were maintained between ST1 and ST2. Oxygen uptake was not significantly different between ST1 and ST2, or conditions. Respiratory exchange ratio (RER) values decreased from 0.98 ± 0.02 in ST1 to 0.91 ± 0.02 in ST2 for PL (*P *= 0.003), supporting reduced total carbohydrate oxidation rates (*P *= 0.007). Mean blood glucose was maintained in CPE across ST trials, and was significantly greater than PL in ST2 (4.77 ± 0.09 mmol.L^-1 ^for CPE compared with 4.18 ± 0.06 mmol.L^-1 ^for PL, *P *< 0.001). Mean distance during PT2 was 2.96 km (or 17.1%) further with CPE than PL (*P *= 0.003). Mean power significantly decreased across PT with PL (134.21 ± 4.79 W and 106.90 ± 3.25 W, respectively; *P *< 0.04).

**Conclusions:**

The use of a CPE beverage improves short-term repeated exercise and subsequent performance compared to PL. Higher rates of carbohydrate oxidation, maintenance of plasma glucose, and decreased levels of fatigue may be beneficial for secondary bouts of performance and faster recovery turnover.

## Background

The introduction of the Nutrition and Health Claims Regulation in 2006 has provided focused guidelines across the European Union for the use of nutrition/health claims, for example "the maintenance of endurance performance" for specific nutrition products. This Regulation aims to ensure that any claim made on foods' labelling, presentation or marketing in the European Union is clear, accurate and based on evidence accepted by the scientific community. Consequently foods or products bearing claims that could mislead consumers may well be eliminated from the market.

As a result, there is increasing interest for sports nutrition product manufacturers to undertake specific research to validate or support marketing claims. VIPER^®^ACTIVE is a specific sports drink produced by Maxinutrition Ltd. The product is a carbohydrate-protein-electrolyte (CPE) formula designed to support exercise performance, energy production, stamina and short term recovery from intense training. The manufacturer guidelines indicate a dosage of 40 g of the product (mixed with 500 ml of water) for use during exercise bouts, equating to an 8.0% concentration (or 7.1% for total carbohydrate).

It is widely established that the ingestion of carbohydrate (CHO) during exercise can improve time to exhaustion [1], through maintenance of plasma glucose concentrations, and increasing total carbohydrate oxidation (CHO_TOT_) rates [2]. Additional evidence exists that by increasing exogenous carbohydrate oxidation (CHO_EXO_) rates [3], beverages containing multiple CHO combinations may have further ergogenic potential [4]. The inclusion of essential electrolytes, namely sodium, into such beverages has also been shown to enhance or support higher hydration levels, or ingestion rates, during and post exercise [5-7].

There has been recent interest in the use of carbohydrate-protein (CP) combinations as a means to not only enhance time to exhaustion compared to a CHO beverage [8], but also to improve post exercise recovery rates. It has been demonstrated [9] that the ingestion of a carbohydrate-casein hydrolysate beverage significantly enhanced late stage cycling time trial performance in comparison to CHO only; and attenuated post exercise creatine kinase concentrations along with subjective muscle soreness. The ergogenic potential of CP beverages firstly appears to be explained by the high CHO ingestion rates of ~60 g.hr^-1^, along with an independent caloric advantage though the co-ingestion of ~20 g.hr^-1 ^of protein. The innovation of nutrient timing has also implied the need for early carbohydrate [10] and/or protein ingestion post exercise [11], particularly when repeated short term training bouts are undertaken. Practical methods to support initial training bouts, as well as short term recovery are therefore warranted, including the assessment of specific formulas which utilise a complete array of essential nutrients, namely combined carbohydrates, essential amino acids and key electrolytes, that may enhance acute and repeated bouts of exercise.

The aim of this study was therefore to undertake an independent assessment of the potential influence of a commercially available CPE beverage (VIPER^®^ACTIVE) on repeated submaximal physiological and work output parameters in comparison to a matched placebo (PL). A further aim was to assess the influence of both beverages on subsequent time trial performance following short term recovery.

## Methods

### Participants

Seventeen healthy males volunteered for participation in the study. Participants were required to be 18-40 years of age, and to be recreationally active. For study inclusion, participants were required to: satisfactorily complete a health screen questionnaire; not be taking any other supplementation; not have dysglycemia or known diabetic conditions; and have a maximal oxygen uptake between 40-59 ml·kg^-1^∙min^-1^. Following a study briefing, all participants provided written, informed consent for inclusion. Ethical approval for the study was provided by the University of Hertfordshire Life Sciences Ethics Committee.

### Preliminary testing

All testing was undertaken in the Human Physiology Laboratory, Division of Sport, Health and Exercise, University of Hertfordshire. Upon entry to the laboratory, nude body mass (Seca 780, Hamburg, Germany) and height were recorded. All participants then performed a maximal oxygen uptake (VO_2max_) test on a Computrainer cycle-ergometer (RaceMate Inc., Seattle, US) to assess against inclusion criteria. After a 5 minute warm-up at a standardised 100 W workload, a continuous ramp protocol (starting at 150 W) was employed with workload increasing at a rate of 15 min^-1^.

Expired air was sampled throughout all tests with an online gas analyser (Metalyser 3B, Cortex Biophysik, Leipzig, Germany) to assess VO_2max _and other respiratory variables. Heart rate (HR) was measured by means of a telemetric system (Polar Electro Oy, Kempele, Finland). Ratings of perceived exertion (RPE) were collected at 1 minute intervals, using the Borg 6-20 subjective exertion scale [12]. The test concluded when participants reached volitional exhaustion or were unable to maintain the required power output.

VO_2max _was defined when a minimum of two of the following criteria were attained: 1) an increase of no more than 2 ml·kg^-1^·min^-1 ^in oxygen consumption with additional workload, 2) attainment of maximal predicted heart rate (± 10 beats.min^-1^) and 3) a respiratory exchange ratio (RER) of > 1.05. Maximal power (W_max_) was calculated by adding the fraction of time spent in the final non-completed workload, multiplied by the 15 W increment, to the final completed workload.

Only one participant did not fulfil the inclusion criteria, and was therefore withdrawn from the experimental study. Remaining participants undertook an habituation trial a week later to confirm the exercise intensity required for the main experiment using the same cycle-ergometer. Participant data are shown in Table 1.

**Table 1:** Summary of participant characteristics and pre-experimental data collection

Age (Years)	Height (m)	Weight (kg)	VO_2max _(L.min^-1^)	VO_2max _(ml.kg^-1^.min^-1^)	W_max _(watts)
19.56 ± 0.89	1.76 ± 0.07	70.05 ± 7.90	3.47 ± 0.49	49.69 ± 4.19	267.38 ± 30.75

Values are presented as mean ± SD; n = 16; VO_2max_, maximal oxygen uptake; W_max_, maximal power output.

### Experimental trials

Experimental trials were conducted under laboratory and temperature controlled conditions (temperature: 20.2 ± 0.5°C; barometric pressure - range: 904-1015 mBar; and relative humidity -range: 24-47%), with no statistically significant differences demonstrated between trials (*P *> 0.05) for any of the environmental variables.

A randomised, double-blind, placebo controlled design was employed, with participants being required to attend the laboratory at the same time of day over two trials (separated by one week). Participants were requested to arrive at the laboratory having overnight fasted (12 hours) and having refrained from strenuous activity for the previous 72 hours. Additionally, individual food diaries for the 72 hours prior to each trial were provided by all subjects to assess for dietary compliance. On arrival to the laboratory, participants were required to complete a subjective muscle soreness questionnaire for the knee extensors and hamstring areas, as well as a daily analysis of life demands for athletes questionnaire (DALDA [13]).

Each trial consisted of two exercise bouts separated by a two hour recovery period. For each exercise bout, participants were required to complete a 45 minute submaximal exercise period (ST), followed immediately by a 45 minute time trial performance test (PT). A standardised warm up of 5 minutes at 100 W on the same Computrainer cycle-ergometer used in pre-testing conditions was employed for all participants prior to each exercise bout. At the end of the warm up period, participants were provided with an opaque drinks bottle containing 500 ml of either the test drink (40 g of a combined dextrose, maltodextrin and hydrolysed whey protein formula (VIPER^®^ACTIVE, Maxinutrition Ltd.) delivering an 8% concentrated solution) or a taste/appearance matched citrus fruit concentrate placebo. A fixed volume of 100 ml was consumed by the participants at 0, 10, 20, 30 and 40 minutes of the submaximal exercise period. The test beverage per 100 g comprised: 7.1 g of protein; 88.4 g of total carbohydrate (of which 50.6 g glucose); 0.4 g of total fat; 0.53 g of sodium; 0.03 g of magnesium; 0.17 g of potassium and 0.14 g of calcium, and delivered 386 kcal. Conversely the placebo beverage per 100 g comprised: 0.6 g of total carbohydrate; 0.2 g of protein; trace amounts of total fat and sodium, and delivered only 8 kcal.

Submaximal exercise (ST1) comprised 45 minutes cycling at a workload equivalent to 60% VO_2max_. During the ST period, capilliarised fingertip blood sampling (100 μl) was undertaken at 10 minute intervals for the assessment of blood lactate and glucose (Biosen C, EKF Diagnostics, Barleben, Germany). Respiratory measurements were ascertained at 10 minute intervals during ST to confirm intensity adherence utilising expired air analysis. RPE and HR measurements were collected at 5 minute intervals. Mean power output (W), speed (km.hr^-1^) and distance covered (km) were also assessed during ST.

On completion of the ST protocol, participants immediately undertook a 45 minute maximal time trial performance test (PT1). Only time elapsed was visible to the participants. No test drinks were administered during PT1. Mean power output (W), speed (km.hr^-1^), distance covered (km), RPE and HR were assessed at 10 minute intervals during PT1.

At the end of the first 90 minute exercise period, participants undertook a 2 hour superivsed recovery period. During this period participants were provided with 500 ml of the test drink at 0 and 60 minutes into recovery. In addition, all participants received a standard protein meal bar (Promax™ Meal Bar, Maxinutrition Ltd.) at 60 minutes into recovery. This was to avoid any unnecessary risks of severe hypoglycaemia occurring during the placebo trial. The standard protein bar comprised 206 kcal, containing 21.6 g of protein, 17.0 g of carbohydrate (of which 9.5 g sugars), 5.7 g of total fat, and 0.05 g of sodium.

At the end of the recovery period, all participants underwent a second exercise period, comprising the same protocol for both submaximal (ST2) and time trial performance (PT2) previously described. Participants returned to the laboratory one week later to complete the same experimental procedure on the alternate drink. On completion of each trial, participants were provided with three muscle soreness/DALDA questionnaires for completion on waking on days 1, 2, and 3.

### Calculations and statistical analyses

Calculation of total carbohydrate (CHO_TOT_) and fat oxidation (FAT_TOT_) rates in g.min^-1 ^were assessed using absolute expired air measurements of VO_2 _and VCO_2 _(L.min^-1^) according to the following stoichiometric equations [14]:

$$\text{CH}{\text{O}}_{\text{TOT}}=\text{ 4}.\text{585 }\cdot \text{ VC}{\text{O}}_{2}-\text{ 3}.\text{226 }\cdot \text{ V}{\text{O}}_{2}$$

$$\text{FA}{\text{T}}_{\text{TOT}}=\text{ 1}.\text{695 }\cdot \text{ V}{\text{O}}_{\text{2}}-\text{ 1}.\text{7}0\text{1 }\cdot \text{ VC}{\text{O}}_{\text{2}}$$

Statistical analyses were performed using SPSS Statistics for Windows version 17 (SPSS, Chicago, USA). A two-way analysis of variance (ANOVA) for repeated measures was used to assess interactions between trial (ST or PT), condition (beverage used) and where applicable, time, for all variables. Where F ratios were found to be significant a Bonferroni post hoc test was applied. An alpha level of 0.05 was employed for assessment of statistical significance. All data are reported as means ± SE.

## Results

### Submaximal exercise trials (ST)

#### Distance, speed and power output

Data for distance covered (km) and average speed output (km.hr^-1^) are represented in Table 2. There was a significant interaction effect for total distance covered during submaximal exercise (F = 8.054; *P *= 0.013). Whereas total distance covered with CPE was not different between trials; there was a significant reduction in mean distance covered with PL (20.18 ± 0.28 km in ST1 v 18.34 ± 0.36 km in ST2; *P *= 0.0001). This represented a 9.12% decrease in submaximal performance with PL. In addition, reduced distance covered in ST2 for the PL condition was specifically noted in the last 15 minutes of the trial (*P *= 0.0001). Accordingly, there was a similar interaction effect for average speed output during submaximal exercise between trials and conditions (F = 8.724; P = 0.010).

**Table 2:** Comparison between test beverages on distance covered and average speed during submaximal exercise trials

	PL		CPE	
	**ST 1**	**ST 2**	**ST 1**	**ST 2**

Total Distance Covered (km)	20.18 ± 0.28	18.34 ± 0.36*	20.03 ± 0.32	19.17 ± 0.44

Distance in last 15 mins(km)	6.65 ± 0.11	5.84 ± 0.16*	6.67 ± 0.12	6.32 ± 0.18

Average Speed (km.hr^-1^)	26.89 ± 0.39	24.67 ± 0.46*	26.54 ± 0.36	25.70 ± 0.56

Average Speed - last 15 mins (km.hr^-1^)	27.05 ± 0.39	24.75 ± 0.49*	26.72 ± 0.43	25.64 ± 0.58

Values are presented as mean ± SE; n = 16; PL, Placebo; CPE, carbohydrate-protein-electrolyte; ST1, submaximal exercise trial 1, ST2, submaximal exercise trial 2. * denotes significant difference (P = 0.0001) between trials within condition only.

**Figure 1:**
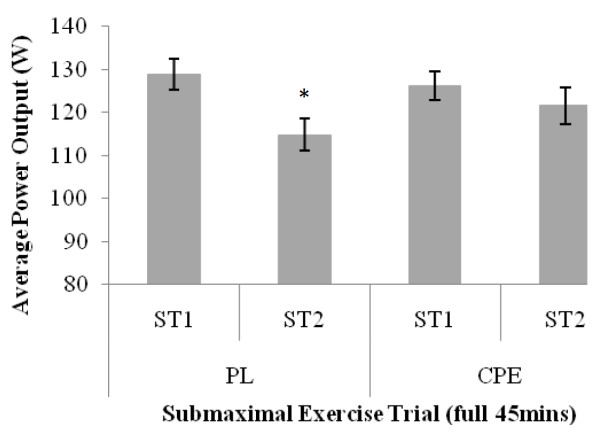
**Assessment of test beverages on average power output watts) during submaximal exercise trials**. Data is presented as mean ± SE; n = 16. PL, Placebo; CPE, carbohydrate-protein-electrolyte; ST1, submaximal exercise trial 1, ST2, submaximal exercise trial 2. * denotes significant difference P = 0.002) between trials within condition only.

**Figure 2:**
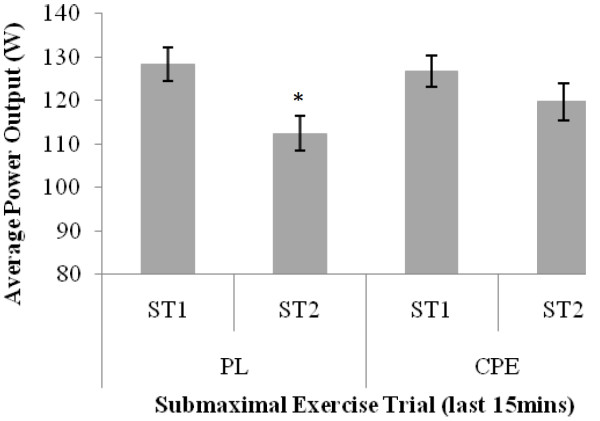
**Assessment of test beverages on average power output watts) during final 15 minutes of submaximal exercise trials**. Data is presented as mean ± SE; n = 16. PL, Placebo; CPE, carbohydrate-protein-electrolyte; ST1, submaximal exercise trial 1, ST2, submaximal exercise trial 2. * denotes significant difference P = 0.0001) between trials within condition only.

Data for average power output are shown in Figures 1 and 2. During submaximal exercise, there was a significant interaction effect for average power output (F = 7.637; *P *= 0.015). Over the full 45 minute trial, power output significantly decreased by 10.9% from 128.89 ± 3.61 W in ST1 to 114.82 ± 4.04 W in ST2 (*P *= 0.002) for PL only. A similar pattern was observed for the last 15 minutes of the exercise trial, with average power output being significantly lower in ST2 (112.38 ± 4.22 W) compared to ST1 (128.38 ± 3.85 W) for PL only (*P *= 0.0001). No significant differences were found for the CPE beverage between trials.

#### Cardio-respiratory and subjective exertion data

Data for submaximal cardiorespiratory variables, total oxidation rates and RPE data are represented in Table 3. No significant differences were found within condition or between trials for oxygen consumption (VO_2_) (*P *> 0.05), demonstrating adherence to the exercise intensity. There was, however, a significant difference between trials for carbon dioxide (VCO_2_) production (F = 18.814; *P *= 0.001). VCO_2 _was significantly lower in ST2 compared to ST1 for PL (1.816 ± 0.076 L.min^-1 ^v 2.031 ± 0.100 L.min^-1^, *P *= 0.0001). There was also a significant difference in mean VCO_2 _in ST2 between PL and CPE (1.816 ± 0.076 L.min^-1 ^v 1.914 ± 0.066 L.min^-1 ^respectively, *P *= 0.029).

**Table 3:** Comparison between test beverages on cardiorespiratory variables, total oxidation rates and subjective exertion data during submaximal exercise trials

	PL		CPE	
	**ST 1**	**ST 2**	**ST 1**	**ST 2**

VO_2 _(L.min^-1^)	2.040 ± 0.058	1.995 ± 0.071	2.062 ± 0.058	2.052 ± 0.071

VCO_2 _(L.min^-1^)	2.031 ± 0.100	1.816 ± 0.076*	2.021 ± 0.064	1.914 ± 0.066**^#^**

RER	0.98 ± 0.02	0.91 ± 0.02*	0.98 ± 0.02	0.94 ± 0.01

CHO_TOT _(g.min^-1^)	2.729 ± 0.328	1.891 ± 0.226*	2.615 ± 0.216	2.159 ± 0.132

FAT_TOT _(g.min^-1^)	0.004 ± 0.108	0.293 ± 0.085*	0.057 ± 0.083	0.221 ± 0.049

V_E _(L.min^-1^)	51.74 ± 2.60	50.39 ± 2.94	47.94 ± 2.16	47.62 ± 2.36**

Heart Rate (b.min^-1^)	136.88 ± 2.73	142.58 ± 3.03*	138.83 ± 2.77	145.39 ± 2.54

RPE (6-20)	11.21 ± 0.43	12.39 ± 0.60	11.46 ± 0.43	11.99 ± 0.52

Values are presented as mean ± SE; n = 16; PL, Placebo; CPE, carbohydrate-protein-electrolyte; ST1, submaximal exercise trial 1, ST2, submaximal exercise trial 2; VO_2_, oxygen consumption; VCO_2_, expired carbon dioxide; RER, respiratory exchange ratio; CHO_TOT_, total carbohydrate oxidation; FAT_TOT_, total fat oxidation; V_E_, minute ventilation; RPE, rating of perceived exertion. * denotes significant difference (P < 0.05) between trials within condition only. # denotes significant difference (P < 0.05) from PL within trial. ** denotes significant difference between conditions overall (P < 0.05).

A significant interaction effect was found for CHO_TOT _across trials (F = 22.407; *P *= 0.0001). With PL, mean CHO_TOT _significantly reduced from 2.729 ± 0.328 g.min^-1 ^in ST1 to 1.891 ± 0.226 g.min^-1 ^in ST2 (*P *= 0.007). Whilst mean CHO_TOT _reduced between submaximal bouts, no significant differences were observed between trials with CPE. Similarly, a significant interaction effect was found for FAT_TOT _across trials (F = 21.330; *P *= 0.0001). Mean FAT_TOT _increased across submaximal exercise bouts, but was only deemed significant with PL (increasing from 0.004 ± 0.108 g.min^-1 ^in ST1 to 0.293 ± 0.085 g.min^-1 ^in ST2; *P *= 0.036).

There was a significant interaction effect found for average heart rate data (F = 25.756; *P *= 0.0001). Despite similar trends between conditions, average heart rate (b.min^-1^) was only significantly elevated in the PL group between trials (*P *= 0.02). No significant differences were reported for RPE data within condition or between trials.

#### Wholeblood data

**Figure 3:**
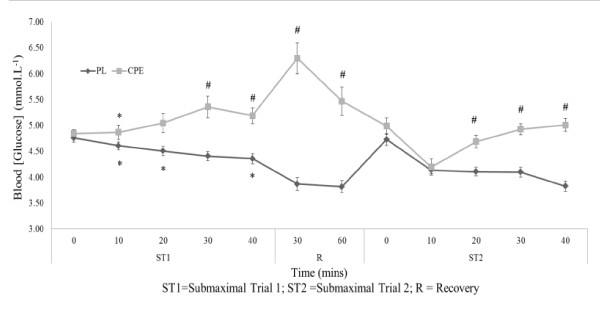
**Assessment of test beverages on blood glucose mmol.L^-1^) during submaximal exercise trials**. Data is presented as mean ± SE; n = 16. PL, Placebo; CPE, carbohydrate-protein-electrolyte; ST1, submaximal exercise trial 1, ST2, submaximal exercise trial 2. * denotes significant difference P < 0.005) between trials within condition only. # denotes significant difference P < 0.008) between conditions within trial.

Data for blood glucose are represented in Figure 3. No significant differences were found between trials or conditions for resting values (*P *= 0.327). There was, however, a significant interaction effect over both time and condition (F = 3.654; *P *= 0.01). Mean blood glucose was significantly greater over the first exercise bout with CPE compared to PL (5.06 ± 0.13 mmol.L^-1 ^and 4.53 ± 0.08 mmol.L^-1 ^respectively; *P *= 0.002).

During recovery between exercise bouts, there was a significant interaction effect (*P *< 0.001), with mean blood glucose being significantly lower with PL both at 30 minutes (6.30 ± 0.30 mmol.L^-1 ^for CPE and 3.87 ± 0.12 mmol.L^-1 ^for PL, *P *< 0.01) and 60 minutes (5.47 ± 0.27 mmol.L^-1 ^for CPE and 3.82 ± 0.12 mmol.L^-1 ^for PL, *P *< 0.01). Mean blood glucose in ST2 was maintained with CPE compared to ST1; and was significantly higher than with PL during ST2 (4.77 ± 0.08 mmol.L^1 ^for CPE compared with 4.18 ± 0.06 mmol.L^-1 ^for PL, *P *< 0.001).

**Figure 4:**
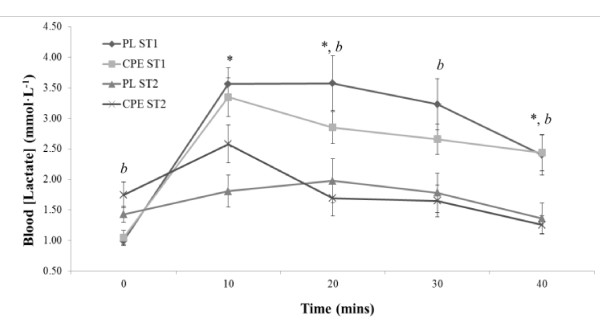
**Assessment of test beverages on blood lactate mmol.L^-1^) during submaximal exercise trials**. Data is presented as mean ± SE; n = 16. PL, Placebo; CPE, carbohydrate-protein-electrolyte; ST1, submaximal exercise trial 1, ST2, submaximal exercise trial 2. * denotes significant difference P < 0.05) between trials within condition only PL). *b *denotes significant difference P < 0.05) between trials within condition only CPE).

Data for blood lactate are represented in Figure 4. Whilst there were no significant differences for resting lactate between conditions, blood lactate was elevated at the beginning of the second exercise bout with CPE compared to the first bout only (1.74 ± 0.21 mmol.L^-1 ^compared to 1.04 ± 0.12 mmol.L^-1^, *P *= 0.04). Mean data demonstrated a significant decrease in blood lactate between exercise bouts for CPE (2.47 ± 0.20 mmol.L^-1 ^compared to 1.78 ± 0.18 mmol.L^-1^, *P *= 0.005) and for PL (2.75 ± 0.26 mmol.L^-1 ^compared to 1.67 ± 0.17 mmol.L^-1^, *P *= 0.009). There were no other significant differences reported between conditions.

### Time trial performance data

**Figure 5:**
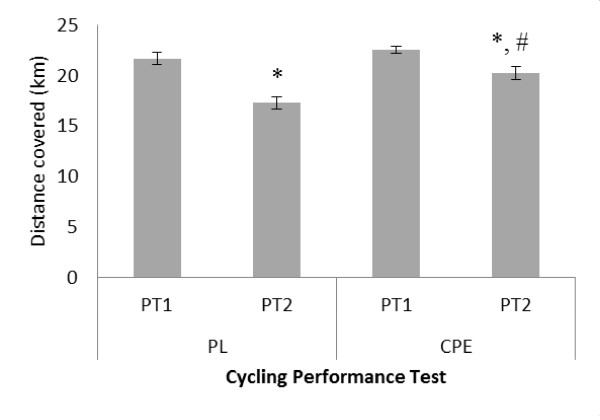
**Assessment of test beverages on total distance covered km) during a 45 minute cycling performance test**. Data is presented as mean ± SE; n = 16. PL, Placebo; CPE, carbohydrate-protein-electrolyte; PT1, performance time trial 1, PT2, performance time trial 2. * denotes significant difference P < 0.05) between trials within condition only.# denotes significant difference from PL within trial P = 0.003).

Data for overall distance covered during the time trial performance tests (PT) are shown in Figure 5. A significant interaction effect was found for total distance covered (F = 12.231; *P *= 0.004). No differences were reported between conditions for PT1. However, with PL, average distance covered fell from 21.64 ± 0.58 km in PT1 to 17.27 ± 0.62 km in PT2 (P = 0.0001), representing a 20.2% reduction in performance. Total distance covered was also lower in PT2 compared to PT1 with CPE (20.23 ± 0.65 km v 22.55 ± 0.34 km respectively; *P *= 0.02), representing a 10.3% reduction in performance. However, there was a significant difference between conditions following PT2, with the CPE group cycling on average 2.96 km further than the PL group (*P *= 0.003) representing a 17.1% difference between conditions.

Additionally, assessment of distance covered in the last 15 minutes of the PT revealed a significant interaction effect (F = 6.288; *P *= 0.024), with mean distance reducing from 7.29 ± 0.21 km to 5.81 ± 0.24 km with PL across trials (*P *= 0.0001), and from 7.76 ± 0.15 km to 6.74 ± 0.25 km with CPE across trials (*P *= 0.007). The reduction observed with PL was 16.0% greater than with CPE for PT2 (*P *= 0.01).

Data for average speed (km.hr^-1^) and power output (W) during the PT tests are shown in Table 4 including data for mean heart rate (b.min^-1^) and RPE. A significant interaction effect was found for average speed (F = 13.486; *P *= 0.003). Whilst data was not different between conditions for PT1, average speed was reduced in PT2 for both conditions (*P *< 0.04). Furthermore, average speed in PT2 was significantly greater by 8.86% with CPE compared to PL (*P *= 0.02). Data was also significantly different over the last 15 minutes of PT2, with average speed being greater by 10.2% with CPE compared to PL (*P *= 0.009). Accordingly, a similar interaction effect was reported for power output (F = 9.660; *P *= 0.008), particularly with regards to a significantly lower average power output reported between PT trials for PL (*P *= 0.0001). At the end of PT2, average power output was 15.9% higher with CPE compared to PL (*P *= 0.008), and 18.8% higher when data for the last 15 minutes was assessed (*P *= 0.004).

**Table 4:** Comparison between test beverages on average speed, power, heart rate and RPE data during a 45 minute cycling performance trial

	PL		CPE	
	**PT 1**	**PT 2**	**PT 1**	**PT 2**

Average speed (km.hr^-1^)	27.52 ± 0.47	23.93 ± 0.45*	27.64 ± 0.41	26.05 ± 0.58*^#^

Average speed (last 15 mins: km.hr^-1^)	27.73 ± 0.50	23.71 ± 0.47*	28.06 ± 0.42	26.14 ± 0.60*^#^

Average power (W)	134.21 ± 4.79	106.90 ± 3.25*	136.82 ± 3.80	123.97 ± 4.42^#^

Average power (last 15 mins: W)	136.70 ± 5.21	105.30 ± 3.18*	141.52 ± 3.99	125.14 ± 4.69^#^

Heart Rate (b.min^-1^)	155.53 ± 4.27	140.34 ± 4.54*	163.84 ± 3.60	153.73 ± 4.45

RPE (6-20)	15.75 ± 0.46	16.45 ± 0.41	15.87 ± 0.45	16.35 ± 0.46^b^

Values are presented as mean ± SE; n = 16; PL, Placebo; CPE, carbohydrate-protein-electrolyte; PT1, performance time trial 1, PT2, performance time trial 2; RPE, rating of perceived exertion. * denotes a significant difference from PT1 within condition (P < 0.04), # denotes a significant difference between conditions within trial (P < 0.02), b denotes a significant difference between trials only (P < 0.05).

A significant interaction effect was found for heart rate data across trials (F = 17.220; *P *= 0.001), with average heart rate shown to be significantly lower in PT2 compared to PT1 for PL only (*P *= 0.005). No other differences were observed for heart rate between trials or conditions. Data for RPE demonstrated consistent hard to very hard exertion across PT tests, and was significantly higher in PT2 compared to PT1 only (F = 4.752; *P *= 0.047). No other differences were observed for RPE within or between conditions.

### Post trial questionnaire and subjective muscle soreness assessment

The DALDA questionnaire is divided into two sections representing factors associated with life stress (part A) and symptoms of stress (part B). No significant differences were found for any of the pre trial DALDA responses (*P *> 0.05). Data for post trial responses are shown in Table 5. No significant interaction effect was found for life stress responses across days or conditions (*P *> 0.05). For part B, whilst a significant interaction effect was demonstrated across days (F = 4.708; *P *= 0.021), post hoc analysis only revealed a trend for lower overall responses on day 3 compared to day 1 (*P *= 0.08).

**Table 5:** Assessment of test beverage influence on post trial DALDA questionnaire and subjective muscle soreness

		PL			CPE	
	**P1**	**P2**	**P3**	**P1**	**P2**	**P3**

DALDA Part A	1.46 ± 0.39	1.08 ± 0.33	0.85 ± 0.27	1.00 ± 0.30	1.15 ± 0.30	1.08 ± 0.24

DALDA Part B	3.08 ± 0.76	3.15 ± 0.94	1.92 ± 0.74	3.23 ± 0.65	2.15 ± 0.59	1.77 ± 0.30

MQS	2.21 ± 0.35	1.65 ± 0.20	1.49 ± 0.16	1.68 ± 0.21	1.36 ± 0.14	1.28 ± 0.15*

MVLS	2.27 ± 0.38	1.62 ± 0.21	1.50 ± 0.16	1.65 ± 0.22	1.35 ± 0.15	1.19 ± 0.13*^#^

MDVLS	2.31 ± 0.35	1.54 ± 0.18	1.46 ± 0.14	1.69 ± 0.26	1.31 ± 0.17	1.15 ± 0.10*^#^

MHS	2.15 ± 0.33	1.69 ± 0.21	1.35 ± 0.13	1.73 ± 0.31	1.58 ± 0.30	1.42 ± 0.21

Values are presented as mean ± SE; n = 16; PL, Placebo; CPE, carbohydrate-protein-electrolyte; P1-3, post trial days 1 to 3; MQS, mean quadriceps soreness; MVLS, mean vastus lateralis soreness; MDVLS, mean distal vastus lateralis soreness; MHS, mean hamstring soreness.* denotes a significant reduction between P1 and P3 overall (P < 0.05). # denotes a significant reduction between P1 and P2 overall (P < 0.05).

No significant differences were found for any of the pre trial muscle soreness assessments (*P *> 0.05). Post trial muscle soreness assessment data are represented in Table 5. Mean quadriceps soreness was significantly different post trial (F = 7.824; *P *= 0.013), with soreness ratios only different between days 1 and 3 (*P *= 0.05). Data was not different between conditions (*P *> 0.05). Likewise, mean vastus lateralis (VL), and mean distal VL soreness assessment was significantly different between days 1 and 2, and 1 and 3 post trial only (*P *< 0.05). No other differences were observed for soreness data.

## Discussion

### Submaximal exercise

One of the key findings from this study was that the ingestion of a CPE beverage maintained total distance, average speed and power output in ST2 when compared to PL. At a prescribed exercise intensity, total distance covered significantly decreased by 9.12% from 20.18 ± 0.28 km in ST1 to 18.34 ± 0.36 km in ST2 when participants consumed a fruit concentrate PL. In contrast, there was no significant difference between ST1 and ST2 for total distance covered when participants consumed a CPE beverage. Whilst there were no differences found between conditions for ST1 or ST2, the significant reduction in work output for the PL group does support previous research indicating that CHO ingestion is likely to be more beneficial for longer duration [15,16] or subsequent high intensity exercise bouts [17].

Additionally, as there has been previous interest in the effect of CHO beverages in the latter stages of exercise, the decrement in exercise maintenance with the ingestion of PL was also observed in the last 15 minutes of ST2. When participants undertook ST2 during the PL condition, average speed significantly reduced from 27.05 ± 0.39 km.hr^-1 ^in ST1 to 24.75 ± 0.49 km.hr^-1 ^in ST2. This was replicated with a significant reduction in average power output in the final 15 minutes of ST2 of 16.0 W in the PL condition. As the degree of statistical significance was greater at 45 minutes compared with 30 minutes, it can be inferred that the level of fatigue was exacerbated in the last 15 minutes without ingestion of CPE.

The maintenance of submaximal work output observed with CPE indicates the beneficial effects of such beverages on single day repeated training sessions. It is probable that such replication of work output is explained by the maintenance of plasma glucose, especially in ST2. Interestingly, the ingestion of CPE resulted in a greater mean blood glucose in the first exercise bout compared with PL (5.06 ± 0.13 mmol.L^-1 ^and 4.53 ± 0.08 mmol.L^-1 ^respectively), but this did not impact on short term work output in ST1. The maintenance of a higher mean blood glucose was further apparent with CPE in ST2 (4.77 ± 0.08 mmol.L^-1 ^compared with 4.18 ± 0.06 mmol.L^-1 ^for PL), which potentially contributed to overall and end stage work output.

The ingestion of a PL beverage clearly resulted in increased levels of fatigue, demonstrated by significant reductions in power output and total distance covered during ST2 relative to ST1. Concomitant reductions in VCO_2_, RER and CHO_TOT _suggest that depletion of endogenous energy stores may be the major mechanism contributing to short term fatigue, particularly in a glycogen-fasted state. With increased utilisation of endogenous carbohydrate, there will be a decreased reliance on glycolytic flux and hence reduced lactic acid production, as demonstrated in the PL condition. With a reduced demand to buffer hydrogen ion production, this likely explains the significantly lowered VCO_2 _levels observed in ST2 for PL. Whilst mean CHO_TOT _was observed to decrease in ST2 with CPE (from 2.615 ± 0.216 g.min^-1 ^in ST1 to 2.159 ± 0.132 g.min^-1 ^in ST1), the reduction was not significant, and indicates a relative maintenance of CHO_TOT _throughout the repeated submaximal exercise. The absolute reduction between submaximal bouts for CHO_TOT _in the CPE trial could be explained by low carbohydrate ingestion rates used in the study.

Whilst CHO_TOT _was not assessed during the recovery period, the inclusion of a double bolus of the test beverage at 0 and 60 minutes of recovery resulted in significant differences in mean blood glucose between conditions at 30 minutes (6.30 ± 0.30 mmol.L^-1 ^for CPE and 3.87 ± 0.12 mmol.L^-1 ^for PL) and 60 minutes (5.47 ± 0.27 mmol.L^-1 ^for CPE and 3.82 ± 0.12 mmol.L^-1 ^for PL) of the recovery period. The inclusion of a standard protein bar at 60 minutes into the recovery period was employed to minimise any absolute risk of hypoglycaemia in the PL condition, and to replicate strategies often employed by athletes in daily practice. In the CPE condition a total of 123.1 g of CHO was therefore ingested prior to the start of ST2 in comparison to 17.7 g ingested with the PL condition. Prior to the start of ST2, this would have equated to a total CHO ingestion rate of 0.59 g.min^-1 ^for the CPE condition. This is considerably below the 1.0-1.2 g.min^-1 ^suggested saturation range of intestinal glucose transporters [16,18], yet still infers an ergogenic benefit.

### Performance exercise

There has been much, and often controversial interest, in the potential performance ergogenic effects of CHO beverages both for shorter duration exercise sessions, as well as repeated bouts. It is widely known that in the absence of sufficient CHO, absolute work output will gradually decline with exercise duration and intensity, based on both liver and muscle glycogen depletion rates, and associated mechanisms of intracellular fatigue. In this study, the use of a CPE beverage did not confer performance advantages in PT1 compared to PL, with average power outputs being comparable (134.21 ± 4.79 W for PL and 136.82 ± 3.80 W for CPE). Interestingly, in PT1, mean distance when consuming CPE was 0.91 km greater than PL, which comprised a 4.2% overall improvement comparable to other studies [19].

The lack of statistical significance between conditions for PT1 however do conflict with other studies both for cycling [20] and running tests [21]. In the latter study, the ingestion of a 6.4% CHO-E solution 30 minutes before and at 15 minute intervals during a 1-hr treadmill run, significantly improved performance by 2.7%. Both studies proposed that the inclusion of carbohydrate prior to exercise resulted in higher CHO_TOT _which conveyed the performance increments in the latter stages of exercise. In the current study, carbohydrate ingestion preceded PT1, but not under resting conditions. The lack of difference in CHO_TOT _between conditions for ST1 suggests that ingestion rates were not of sufficient magnitude to elicit short term performance gains. In the previous study [21], participants ingested a total of 67.1 g of CHO prior to completion of a time trial (effectively an ingestion rate of 0.75 g.min^-1^). In the current study, participants ingested a total of 35.4 g CHO prior to completion of PT1 (an effective ingestion rate of 0.39 g.min^-1^). It is therefore possible that higher ingestion rates either pre exercise and/or during PT1 may have resulted in significant short term gains.

However, when repeated bouts of exercise are undertaken, the beneficial effects of CPE ingestion appear to be more pronounced. Total distance covered in PT2 was 17.1% greater with the ingestion of CPE compared to PL. The demanding nature of the trials was observed, with a significant 10.3% reduction in total distance covered between trials for the CPE condition (22.55 ± 0.34 km for PT1 compared to 20.23 ± 0.65 km in PT2), potentially explained by the relatively low total CHO ingestion rates employed.

Average power output during (and in the final 15 minutes) of PT2 were significantly reduced in PL, demonstrating the contrasting benefits of CPE. Whilst the type and quantity of CHO has been shown to enhance exogenous CHO oxidation rates [3,7,18], late stage performance enhancement may still occur with more conservative ingestion rates. By the start of PT2, during the CPE trial, participants had consumed a total of 158.5 g CHO or 37.3 g.hr^-1^. Comparable ingestion rates have been shown to enhance late stage exercise performance elsewhere [22] despite being below known optimal delivery rates of 1-1.2 g.min^-1 ^or 60-70 g.hr^-1 ^[16].

It is most likely that any ergogenic or recovery effects from the CPE beverage are explained by the combination of the maltodextrin and dextrose formulation. It has been demonstrated that the inclusion of multiple carbohydrates will result in higher exogenous carbohydrate oxidation (CHO_EXO_) rates [23]. The combined uptake of total sugars from the sodium dependent glucose transporter (SGLT1) and GLUT5 intestinal transport mechanisms provides potential for maximal exogenous oxidation rates [3]. Whilst the oxidation rates of both dextrose and maltodextrin are similar, the inclusion of maltodextrin reduces beverage osmolarity, hence increasing the potential for carbohydrate delivery to the intestinal lumen, as well as fluid uptake.

Furthermore, the inclusion of sodium to the test beverage is known to enhance carbohydrate bioavailability [24]. Despite relatively low CHO ingestion rates employed in the current study, an enhancement in both CHO delivery and CHO_EXO _would still have a resultant sparing or even suppressing effect on endogenous CHO utilisation [25], as well as maintaining the CHO_TOT _observed between performance bouts. As CHO_EXO _rates have typically been shown to plateau after 90 minutes of steady state exercise, this in part explains the ergogenic potential observed in PT2 with CPE.

Alternatively, as CHO ingestion rates were below optimal delivery levels, it is possible that the co-ingestion of protein may have provided additional ergogenic value through increased caloric content. Whilst it has been suggested the addition of approximately 2% protein to a CHO beverage has minimal effect on subsequent performance, or glycogen resynthesis [26,27], other studies have demonstrated a positive effect of co-ingestion of protein on endurance performance [8,9,28,29] and short term recovery [30]. When carbohydrate-protein beverages have been administered during acute recovery (in comparison to an iso-energetic carbohydrate beverage), there is supporting evidence that the addition of protein positively enhances repeated same day time to exhaustion trials [31,32]. The most likely explanation for this is the higher caloric content of the beverages employed, in comparison to lower dose carbohydrate only beverages [32]. In the current study, as the protein intake for CPE was only 0.6% or 2.84 g per 40 g serve, any enhancement of acute recovery through insulin-mediated pathways would most likely be explained via the inclusion of a standard protein bar between exercise trials.

In terms of short term recovery post trials, the only significant observations from this study were reductions in mean quadriceps soreness, mean vastus lateralis soreness and mean distal vastus lateralis soreness by day 3. This was expected considering subjects had a 7 day rest period between trials, hence explaining the gradual reduction in perceived soreness for both conditions. As no differences were found between conditions for post exercise muscle soreness or DALDA responses, the inclusion of early protein feeding (mainly in the form of a protein meal bar) may have assisted recovery in both conditions, as demonstrated elsewhere [33]. It has been suggested that the inclusion of protein to a carbohydrate beverage during early recovery, particularly in higher dosages than the present study, may facilitate intracellular rps6 and mTor signalling pathways leading to enhanced protein resynthesis and hence recovery [34-36]. However, beneficial effects of such beverages on acute glycogen resynthesis is most likely accounted for by underlying carbohydrate dosage and content [37].

## Conclusions

In conclusion, the ingestion of commercially available CPE beverage, significantly impacted on both repeated submaximal exercise and cycling time trial performance in comparison to PL. Through maintenance of blood glucose concentrations and CHO_TOT_, the potential sparing of endogenous energy stores supports the inclusion of a CPE beverage for ergogenic benefits. Such beverages may be particularly relevant where recovery between bouts of exercise is relatively short and/or glycogen depletion may significantly increase levels of fatigue.

## Abbreviations

CHO: Carbohydrate; CHO_TOT_: Total carbohydrate oxidation rate (measured in g.min^-1^); CHO_EXO_: Exogenous carbohydrate oxidation rate (measured in g.min^-1^); CPE: Carbohydrate-protein-electrolyte beverage used for the study; FAT_TOT_: Total fat oxidation rate (measured in g.min^-1^); GLUT5: Glucose transporter 5: PL: Placebo beverage used in the beverage; PT: Performance trial; a 1 after denotes first test: 2 denotes second test; RER: Respiratory exchange ratio: the ratio from dividing expired carbon dioxide with oxygen uptake; RPE: Rating of perceived exertion; SGLT1: Sodium dependent glucose transporter 1; ST: Submaximal exercise trial; a 1 after denotes first test: 2 denotes second test; V_E_: Minute ventilation: the amount of air breathed in one minute (L.min^-1^); VO_2_: Volume of oxygen uptake (measured in L.min^-1^); VO_2max_: Maximal oxygen uptake (measured in L.min^-1^) VCO_2_: Volume of expired carbon dioxide (measured in L.min^-1^).

## Competing interests

Design input and funding to support this study was received from Maxinutrition Ltd. Data was collected, analysed and reported independently of the company. No suggestions, pressure or duress were placed on the investigatory team whatsoever.

## Authors' contributions

All authors were involved in the study. JDR was principal researcher, involved with liason with the company, participant screening, beverage assignment, data collection, statistical analysis and report generation; MDT was co-researcher involved with cohort organization, data collection and blood analyses, confirmation of statistical analyses, and helped to draft the manuscript; LSK was involved with monitoring of data collection including collation of performance data, and test beverage administration, as well as overview and editing of manuscript; MGR was involved in quality control, part data collection, and technical accuracy in preparation of the manuscript. All authors read and approved the final manuscript.
